# Should Countries Set an Explicit Health Benefits Package? The Case of the English National Health Service^☆^

**DOI:** 10.1016/j.jval.2016.01.004

**Published:** 2017-01

**Authors:** Peter C Smith, Kalipso Chalkidou

**Affiliations:** 1 Imperial College Business School, London, UK; 2 Institute for Global Health Innovation, Imperial College, London, UK

**Keywords:** cost-effectiveness analysis, health benefits package, universal health coverage

## Abstract

**Background:**

A fundamental debate in the transition towards universal health coverage concerns whether to establish an explicit health benefits package to which all citizens are entitled, and the level of detail in which to specify that package. At one extreme, the treatments to be funded, and the circumstances in which patients qualify for the treatment, might be specified in great detail, and be entirely mandatory. This would make clinicians little more than automata, carrying out prescribed practice. At the other extreme, priorities may be expressed in very broad terms, with no compulsion or other incentives to encourage adherence.

**Objectives:**

The paper examines the arguments for and against setting an explicit benefits package, and discusses the circumstances in which increased detail in specification are most appropriate.

**Methods:**

The English National Health Service is used as a case study, based on institutional history, official documents and research literature.

**Results:**

Although the English NHS does not explicitly specify a health benefits package, it is in some respects establishing an ‘intelligent’ package, based on instruments such as an essential medicines list, clinical guidelines, provider payment and performance reporting, which acknowledges gaps in evidence and variations in local resource constraints.

**Conclusions:**

Further moves towards a more explicit specification are likely to yield substantial benefits in most health systems. Considerations in determining the ‘hardness’ of benefits package specification might include the quality of information about the costs and benefits of treatments, the heterogeneity of patient needs and preferences, the financing regime in place, and the nature of supply side constraints.

## Introduction

The World Health Organization identifies three dimensions of policymaking choices as countries seek to implement universal health coverage (UHC): the groups in the population to be covered, the level of financial protection offered when seeking access to services, and the range of services to be covered. Of these, the first two dimensions frequently offer little realistic scope for policy variation. Allowing access only to certain population subgroups contradicts the fundamental intent of universality. And imposing any level of user charges may exclude access for the poorest groups, as well as entail administrative complexity. Therefore, the central focus of policy will usually be the third dimension of the UHC design: the range of services to be made available, usually referred to as the health benefits package (HBP).

Many high-income countries have sought to maintain packages that are quite comprehensive, in the sense that most clinically accepted interventions have been included [1]. In contrast, low- and middle-income countries with slender resources have been forced to confront the issue of which interventions or services to include in their benefits package [2]. Sometimes, as, for example, in the case of Chile [3] or Mexico [4], this problem has been addressed directly, and a carefully circumscribed package has been explicitly defined. More often, however, the package has been developed piecemeal and implicitly, as, for example, in India [5].

Numerous techniques and processes have been adopted for selecting the benefits package [6]. Nevertheless, whatever the resources available, policymakers will usually wish to maximize the effectiveness of their UHC policy, in the form of maximizing the “value” (however defined) of the health services purchased with the limited publicly funded budget. Economists have advocated the use of cost-effectiveness analysis (CEA) as making this principle operational, on the assumption that the objective to be maximized is health gain. Although the application of the cost-effectiveness criterion suffers from some theoretical limitations, it has enjoyed widespread acceptance as a reasonable principle for prioritizing the use of scarce health service resources [7].

CEA should therefore be an important tool for determining which health services to fund as countries seek to implement UHC. Nevertheless, implementation of the cost-effectiveness criterion for setting the HBP is seriously hampered by major practical limitations, such as the following:

1. Lack of adequate data for many, if not most, interventions;
2. System constraints that preclude immediate changes in service delivery;
3. Political constraints that circumscribe many choices; and
4. Lack of adequate capacity for assembling and synthesizing relevant analytic material [8].

As a result, most benefits packages have been developed in an ad-hoc fashion, sometimes shaped by CEA, but often tempered by practicality and inertia.

The English National Health Service (NHS) is an archetypal central planning approach toward UHC. Furthermore, it has established a renowned agency for assessing new and existing treatments, in the form of the National Institute for Health and Care Excellence (NICE). Yet, notwithstanding the apparent clarity of the NICE terms of reference and the technical resources at its disposal, it has focused mainly on the evaluation of new technologies, and it can be argued that NICE has had only a modest impact on the total range of services actually made available to NHS patients. The English approach toward setting the benefits package is therefore of particular interest as a basis for discussing the tensions and constraints that arise when seeking to determine what health treatments are to be made available [9].

This article examines the extent to which the English NHS has an explicit HBP. It first sets out the arguments for and against such explicitness. It then specifically examines the role of CEA in guiding the creation of the package. A short outline of the English health system then follows, with an assessment of the extent to which that system yields an explicit statement of entitlements. The article then concludes with some general observations on setting the HBP.

## Arguments for and against Setting an Explicit HBP

As documented by Glassman et al. [10]*,* there are numerous well-rehearsed arguments in favor of setting an explicit HBP to which all beneficiaries are entitled:

1. It creates *explicit entitlements* for patients, whose access to services might otherwise be largely determined by clinical professionals, with the consequent potential for arbitrary variations in access.
2. It helps to identify whether *funds are being spent wisely* on services that create the maximum benefit for the society.
3. By specifying the services to be delivered, it facilitates important *resource allocation decisions*, such as regional funding allocations, and other planning functions, creating a precondition for reducing variations in care and outcomes.
4. It facilitates orderly *adherence to budget limits*, which might otherwise be attained only through arbitrary restrictions on access and services.
5. It reduces the risk that providers will require *informal payments* from patients to secure access to high-value services.
6. The entitlements created empower *poor and marginalized groups*, who cannot be made aware of any specific entitlement without an explicit HBP.
7. It creates the preconditions for a market in *complementary health insurance* for services not covered, with a number of potential benefits for the health system as a whole.

It is important to distinguish between explicitness in stating the contents of the benefits package and consistency and rigor in selecting the contents. It is quite conceivable that a package may be made explicit, but the process for selecting the contents is opaque and inconsistent. Some of the aforementioned virtues of an explicit package arise whatever may be the selection process. Nevertheless, most can have full effect only if the package is selected using consistent application of an explicit set of criteria.

Notwithstanding the powerful reasons for developing an explicit benefits package, and basing it on consistent stated criteria, there are also reasons for caution in pursuing an explicitly delineated package:

1. There are very significant *practical difficulties of specifying a package* in enough detail to have an impact on clinicians. Although it may be feasible to make broad statements regarding the services to be delivered, it may be impractical to specify the circumstances in which specific treatments may be funded. This may be because of a lack of suitable evidence and analytic capacity, a lack of adequate information systems or funding mechanisms, or a lack of detailed clinical guidelines on what constitutes best care.
2. A closely defined package may *inhibit innovation*, especially if it is based on treatments to be delivered rather than on disease categories. If the package is not constantly reviewed and updated, there is a risk that it will reflect outdated approaches to care, and ignore new, more efficient treatments or modes of delivering care, and inhibit take-up of those new approaches.
3. In the same vein, the package may *inhibit warranted variations in treatment* that reflect patients’ circumstances or preferences. The contents of any package will be based on broad average responses to treatment in the population at risk. Although it is important that all treatments should be cost-effective, there will often be circumstances in which clinical judgment may suggest departures from usual treatment for specific patients that improve cost-effectiveness. In principle, any package should be flexible enough to accommodate such departures.
4. Explicit statements of patient entitlement may *create serious political and legal difficulties* for health ministries, by appearing to favor certain groups at the expense of others, and giving rise to a sense that some health care is being “rationed.” Of course, the prime reason for the limitations to care is created by the limited budget made available, but that may not be the focus of political debate. In such circumstances, however, a ministry may be prepared to sacrifice improved efficiency by retaining some ambiguity about the nature of the package.
5. There may be *existing rigidities in health system* that preclude moving toward a new package of care. The transition may require investment costs in new infrastructure and training, may cause disruption to the care of existing patients, may entail disinvestment from some services, and may require political and clinical leadership. Furthermore, there may be an asymmetry between the willingness to pay for new programs compared with established programs [11]. Such concerns are particularly important when the innovation has a substantial budgetary impact [12]. They are common to most health system reforms and require careful planning to ensure a smooth transition over time toward the new arrangements.
6. A benefits package may create an *uncertain financial liability* for the health system. By creating entitlements to care, it becomes impossible to limit access through waiting times, user fees, or other informal means. Although this is of course the intention behind creating the package, it may mean that the impact on the UHC budget is uncertain, and if not underpinned by good analysis, the sustainability of the UHC program may be compromised.

In considering these arguments, it is important to distinguish between services that are promised (stated perhaps as entitlements) and services that are actually received by patients. The performance of any health system should be assessed with respect to the scope and quality of services actually received by patients. The specification of a benefits package is merely an instrument for specifying desired levels of attainment, which may or may not be translated into services received. The effectiveness with which an explicit benefits package leads to the right services being delivered to the right patients at the right time with a high level of quality should be the performance measure for an HBP.

## The Role of CEA

The principle of CEA is that, subject to a number of important assumptions, health care interventions can be ranked on the basis of their incremental costs relative to their incremental benefits. This characterization is a severe simplification because it makes a number of assumptions, such as independence of the various treatments under consideration and constant returns to scale. Relaxing these assumptions does not preclude the use of CEA, but does give rise to methodological complexity that is not germane to this article. Benefits are usually measured in terms of expected health gain, although alternative formulations can be envisaged. This leads to a policy prescription of including treatments in the HBP in order of increasing cost-effectiveness, until the available budget is exhausted. An equivalent formulation is to accept for inclusion only those interventions that lie below some cost-effectiveness threshold, the value of which depends on the size of the budget available. Although CEA can be applied to the choices of individuals in a private health insurance market, its usual application is to collectively funded insurance systems, of the sort used to promote universal health coverage [7]. The CEA principle seeks to maximize “value” (expressed in the form of expected health gain) secured by the budget. The “marginal” treatment just included in the package determines the system’s cost-effectiveness threshold [13].

This simple theory has proved robust to methodological challenge, and forms the basis of a great deal of health technology assessments undertaken around the world. Although there are alternative formulations, it has proved difficult to challenge the principle of seeking to maximize the social value secured from the limited budget available. The CEA threshold offers a consistent benchmark for assessing the competing claims of patients on a limited budget, and applies a widely accepted principle of fairness—that those who can benefit the most from health service spending should have priority. A particularly common challenge to the CEA rule has been the suggestion that it should reflect different levels of priority for health gain for different social groups (perhaps weighting gains for disadvantaged groups more highly than others). This modification is potentially important for the groups affected, and creates considerable analytic complexity, but it does not materially affect the principles underlying CEA [14].

The use of CEA has predominantly been in the field of health technology assessment, for setting broad priorities on which treatments should in general be included in the HBP. In doing so, the usual practice is to base CEA on expected average responses to the treatment under scrutiny in the population expected to receive the treatment. Yet if the principle of CEA is accepted, it should in theory extend to every treatment decision made in the health system. That is, every treatment choice offered to patients should be such that the expected cost-effectiveness ratio lies below the health system threshold. The treatment can be offered only when the costs of treatment *for that specific patient* are compared with the expected health gain for that patient [15]. The heterogeneity of patients means that, for many treatments, there may be substantial variation from the average cost-effectiveness ratio. In principle, the treatment should be offered only to those patients for whom that ratio is expected to be less than the threshold [16]. If this rule is breached, the resources spent on the high-cost (or low-benefit) patients could be better spent somewhere else in the health system. In practice, there has been little use of appropriateness criteria in CEA. Once a treatment is accepted in a benefits package, it is usually the case that all patients are entitled to the treatment, even those for whom the potential health gain is low relative to the cost of treatment.

An important reason for failing to apply cost-effectiveness criteria to individual treatment decisions relates to practicality. Many CEA models are based on limited information, and the disaggregate evidence necessary to determine which subgroups should receive the treatment is often absent or inadequate. Moreover, it is difficult to envisage how clinicians could practically discriminate between patients unless they have solid evidence with which to justify their decision. Their training and professional outlook require a focus on the benefits and costs of treatment for individual patients rather than a broader societal perspective. Cost-effectiveness criteria could, in principle, be built into clinical guidelines, specifying the conditions under which specified treatments satisfy the criteria for acceptance. Nevertheless, in practice it has proved difficult to incorporate detailed economic criteria into clinical guidelines. It is also important to note the ethical and political difficulties of discriminating between patient types when making a treatment available [17]. For example, age may often be an important predictor of health benefits from treatment, and so might in principle be included in guidance. Nevertheless, precluding some patients from access to treatment on the grounds of age could be interpreted as discriminatory under equality laws. Such considerations reflect a sort of equity constraint.

Indeed, in contrast to the highly detailed specifications suggested in the preceding paragraphs, it is often the case that the HBP is set in very broad terms, such as requiring provision of “all necessary hospital treatment free of charge” as in Denmark [18]. This may occur when limitations of data, governance, or policy capacity preclude a more refined specification of priorities. In the extreme, priority setting might take the form of financing certain classes of providers (such as public sector hospitals), without reference to the specific treatments they are expected to provide. This was historically the approach toward public sector provision of services in many lower-income countries [19]. The risk of adopting such a broad definition of priorities is that the prioritized providers may provide some services that are not cost-effective (or conversely the nonprioritized sectors may fail to provide services that are cost-effective). Furthermore, because of the funding model, treatments may be provided in inappropriate settings and quality of care may be poor. These failures of efficiency and effectiveness represent an opportunity cost of lacking the capacity to adopt more detailed prioritization.

One final limitation that applies to any priority-setting process, of whatever sort, relates to the issue of enforcement. There is widespread evidence that stated priorities, whether based on CEA or otherwise, are ignored by many health service providers [20]. This may be because priorities are merely advisory and can be ignored with impunity, or because other policy instruments—such as financing mechanisms or regulations—are not aligned with priorities, or because providers are unaware of these priorities. Furthermore, it may be the case that system constraints preclude implementation, or make its costs substantially higher than those assumed when formulating the priorities [21]. In particular, CEA usually considers only long-run average costs, and not the immediate costs of implementation, which may, for example, include retraining of personnel. To some extent, the freedom of providers to depart from a stated health basket may be desirable if they are responding to patient heterogeneity, as discussed earlier. However, the departures may lead to inefficiency if they are unwarranted by patient characteristics, and the consequent treatment is not cost-effective.

It has therefore in practice proved difficult for health systems to apply the principle of cost-effectiveness consistently, comprehensively, or effectively to its insurance coverage decisions. Departures from stated priorities may be warranted when uncertainty is high or evidence lacking. Departures, however, may also result in inefficient use of health system funding if they lead to provision of services that are not cost-effective.

## NICE and the English NHS

### The Institutional Framework

The English NHS is an archetypal centrally planned health system. It is funded overwhelmingly by national taxation, as part of the health ministry’s annual budget. Since its establishment in 1948, the NHS has been subject to numerous structural reforms that have left its basic structure intact. For the purpose of this discussion, the key elements are as follows:

1. A central role for the national ministry in setting policy, monitoring performance, and allocating funding to geographical localities; some of these activities have recently been transferred to a national agency, NHS England, which is responsible for day-to-day operations of the NHS.
2. The NHS Constitution, which sets out a quasi-legal set of rights and responsibilities for citizens concerning access to the NHS.
3. Local health authorities in receipt of an annual budget and responsible for organizing and purchasing the NHS in their locality, a function known as “commissioning”; at present these authorities are called clinical commissioning groups (CCGs) and each is responsible for a population of about 250,000 people.
4. General practitioners (GPs), who are primary care physicians organized into practices serving on average about 8000 people; GPs play an important gatekeeping role. All citizens must register with a GP, and no one can secure access to nonemergency secondary care without a referral by a GP.
5. User charges are either absent or very low.

By international standards, the NHS as a system has low levels of spending, offers high levels of protection from the financial consequences of ill health, and enjoys high levels of popularity among its citizens [22]. In some clinical areas, however, it secures only moderate quality outcomes for its population, relative to other high-income countries. For example, improvements in 5-year cancer survival rates lag behind those found in many other developed countries [23].

### NICE and the Role of CEA

NICE, which was established in 1999, is an important element of the English NHS. Its role is to provide national guidance and advice to improve health and social care, as summarized in Table 1.

The establishment of NICE, and the high public profile it enjoys, is a strong signal that the principles of cost-effectiveness are accepted as an important criterion for the inclusion of health treatments in the English HBP. Although NICE emphasizes that it is not the only relevant criterion, econometric analysis confirms that cost-effectiveness dominates other quantifiable considerations when determining its health technology appraisal decisions [24]. It is noteworthy that policymakers found it necessary to create a “cancer drugs fund” to include high-cost cancer drugs that would not normally have secured approval from NICE on cost-effectiveness grounds [25]. The implication is that certain patients with cancer warranted privileged access to NHS treatments for reasons that go beyond the conventions of routine NICE appraisals [26]. In the same vein, the cost-effectiveness criterion has been relaxed for certain “end-of-life” treatments [27].

One aspect of the NICE guidance on technology appraisals is supported by statute. If NICE produces guidance on a technology appraisal to say that a new medicine should be made available to NHS patients who meet particular criteria, then the local health organizations that are responsible for providing funding for that treatment are under a statutory obligation to ensure that the technology “is, from a date not later than three months from the date of that Technology Appraisal Guidance, normally available” [28]. This process forms the basis for what is in effect an English “essential medicines list.”

As discussed by Mason [29], legislation defines broad categories of health care service that can be provided by the NHS. The statutes, however, are framed within a vague definition of health need characterized by a “reasonable requirement” and by the right to take into account NHS financial capacity. In practice, this means that patients have no entitlement to specific services. The NHS Constitution [30] sets out a patient’s rights including “the right to receive care and treatment that is appropriate to you, meets your needs and reflects your preferences.” Although certain principles and quality criteria are set out, it includes few explicit treatment entitlements, other than drugs approved by NICE and national vaccination and screening programs. It states that “you have the right to expect local decisions on funding of other drugs and treatments to be made rationally following a proper consideration of the evidence. If the local NHS decides not to fund a drug or treatment you and your doctor feel would be right for you, they will explain that decision to you.”

NICE has tried over the years to develop a library of quality indicators, drawn at large from its own clinical guidelines, technology appraisals, and, more recently, public health guidance. The intention is for these to inform pay-for-performance schemes for primary and secondary care providers and commissioners and also for regulation purposes. The use of such regulatory, contractual, and payment mechanisms suggests that the NHS is attempting more clearly to specify services, as opposed to a negative or positive list of medical technologies. For example, the 2012 Health and Social Care Act explicitly requires the Secretary of State to take account of NICE quality standards when discharging his or her duty to improve care quality across the NHS. Reference to “aspirational but achievable” statements of good quality care, increasingly linked to measures of performance for the NHS such as its Outcomes Framework, may represent a move toward more explicit specification of what the NHS in England seeks to offer its users [31].

### Local Management and Decision Making

The management of the NHS depends heavily on about 200 local “commissioners” of care, each of which covers a geographically defined population of about 250,000 people. These CCGs are given fixed budgets with which to purchase routine local services and drugs, and have a strong managerial focus on adherence to those budgets as well as quality of care. The NHS has for many decades used a sophisticated set of formulae to allocate funds to CCGs (and their predecessor organizations), with the intention of securing equal access to services for equal clinical need [32]. In principle, the budget allocated to localities should reflect the costs of providing a stated HBP. In practice, because no such HBP exists, the funding formula is conservative, reflecting expected expenditure on average across England, given the locality’s demographic, health, social, and economic circumstances. A major policy concern is therefore the extent to which the present formulae (which are based on an empirical analysis of previous spending patterns) reflect “unmet” medical needs that the NHS has historically failed to satisfy. Of course, by the same token, the empirical analysis may also reflect some aspects of unwarranted expenditure, in the form of inefficient care or inappropriate treatments.

Although CCGs are subject to many performance criteria, including attainment under the NHS Outcomes Framework, their overriding imperative (and statutory duty) is to “ensure expenditure in a financial year does not exceed the allocated budget” [33]. Therefore, if clinical needs exceed those assumed in the budget (or if there is local inefficiency), there will often be strong pressure on the CCGs to limit access to certain treatments, a freedom they can to some extent exercise under the statutory framework for the NHS. Note also that, depending on existing operational constraints, the local opportunity costs of providing treatments may differ substantially from the national averages assumed by NICE and other central agencies, at least in the short run. Moreover, many commissioners have put in place “exceptional case” panels to consider requests for treatments not usually available to NHS patients [34]. The local discretion on funding decisions has led to a phenomenon known as the NHS “postcode lottery,” a term adopted by the media and politicians when referring to different decisions made by local health care commissioners on whether or not to fund specific health treatments. Examples include variations in drugs made available (even when approved by NICE) [20] and variations in clinical treatment thresholds for treatments such as hip replacement and cataract surgery [35].

There is considerable evidence that clinical variation is widespread, as documented in some detail in the *NHS Atlas of Variation in Healthcare* [36]. Some of these clinical variations can be attributed to differences in local policies. Nevertheless, there also appears to be considerable variation in professional practice [36]. For example, the *Atlas* shows a twofold variation in the use of colonoscopy and flexisigmoidoscopy procedures, after adjustment for age and sex—such clinical variations are quite typical. In some disease areas, efforts have been made to address this through the promulgation of “National Service Frameworks” and other clinical guidelines. These, however, are advisory and have variable levels of effectiveness. Furthermore, as discussed earlier, there will sometimes be good reasons for variation from standard practice. Nevertheless, it remains the case that there is likely to be considerable unwarranted, and therefore inefficient, variation in clinical practice in the NHS, even after allowing for natural variability in demand for services [36].

Thus, notwithstanding the extensive and painstaking research underlying NICE recommendations, the benefits package offered by the English NHS “is arrived at implicitly, as a result of decisions made by national, regional and local decision makers, working within a context of laws, duties, policies, budgets and financial incentives that change over time” [37]. Even though there is an apparent policy desire, through the creation and development of NICE, to create a consistently and comprehensively specified HBP, in practice the English NHS does not have an explicitly specified basket of treatments. This is readily observed through the wide geographical variations in both the availability of and eligibility criteria for many services.

## Conclusions

Rumbold et al. [37] summarize the institutions that shape the NHS benefits package, and identify six broad influences:

1. *Legal and quasi-legal duties.* These are generally vague in content, although the NHS Constitution and requirements to adhere to NICE technology appraisals offer exceptions to this general rule.
2. *NICE.* Increasingly the guidelines and performance metrics being developed by NICE are becoming de facto statements of preferred services, and are extending much beyond the original territory of new technologies.
3. *Government policy.* The NHS is a heavily centrally planned organization in which a multitude of service requirements, guidance, and performance metrics are applied, intended to influence the nature of local service delivery.
4. *National commissioning.* Many specialized services and much of primary care are commissioned by the national leadership body, NHS England.
5. *Local commissioning.* Local CCGs have considerable freedom to apply their own interpretation to national guidance, in the light of local medical needs, service infrastructure, and budget constraints.
6. *Local clinical decisions.* As in all health systems, a great deal of NHS resource allocation arises for the decisions made by clinicians in their face-to-face contact with patients.

Thus, many of the decisions made at more local levels are likely to be influenced (but not determined) by the guidance and requirements put forward by other levels of the system. In practice, this mixture of influences leads to considerable variation in the HBP made available to identical patients using different providers or living in different parts of England. As we have discussed, these variations may to some extent be efficient, and may also be a necessary requirement to secure strict expenditure control within the system. They, however, lead to the firm conclusion that the NHS does not have an explicit HBP. Rather, it has a flexible package, informed by overriding principles, such as equal access and cost-effectiveness, but permitting considerable local flexibility for both commissioners and clinicians.

In the “Introduction” we listed seven benefits of setting an explicit HBP. Several policy problems in the NHS can therefore be attributed, at least in part, to the absence of an explicit HBP. Some of the specific problems are as follows:

1. *Explicit entitlements*. There are few firm entitlements to treatment for patients, and access to many services relies on the variable policies of local commissioners and practitioners.
2. *Spending funds wisely*. It is very difficult to determine whether commissioners are spending their funds in a way that maximizes health benefits for society.
3. *Resource allocation decisions*. NICE is making an increasingly important contribution to the improvement of allocative efficiency in the NHS at the level of individual treatments. Nevertheless, geographical resource allocation decisions are hampered by a lack of an explicit statement of the services to be provided.
4. *Adherence to budget limits.* Budget discipline is strong by international standards. It is, however, achieved by various somewhat arbitrary means, and the lack of an HBP has led to a “postcode” lottery of access to some services.
5. *Informal payments*. With a few exceptions, providers do not require either formal or informal payments from patients in the NHS. Nevertheless, local limitations to access for some services may encourage patients to seek private care, where they can afford it, which must be funded from voluntary private insurance or out-of-pocket.
6. *Poor and marginalized groups.* There is considerable evidence that poor access to services is greatest among disadvantaged social groups, a problem that an explicit HBP may help to address [38].
7. *Complementary health insurance.* There is no significant market in complementary health insurance for services not covered by the NHS. Instead, there is a small but significant market for duplicate private insurance, which seeks to bypass services provided by the NHS through lower waiting times and enhanced convenience. This market has the potential to undermine support for the NHS if it attracts larger numbers of richer people.

Nevertheless, rather than suggesting an absence of an HBP, the English example more accurately highlights variations in the “hardness” of the HBP specification. At one extreme, NICE technology appraisals rule in (or rule out) specific technologies in their entirety (albeit sometimes possibly limited to certain patient subgroups) without reference to local provider circumstances. At the other extreme, a mass of clinical guidelines offers clinical recommendations based on existing evidence, but offer no direct incentives to comply. At an intermediate level, there are clinical practices that are embodied in performance measures that suggest good practice, and that may indirectly therefore affect provider reputation and revenue. They, however, do not directly contain statements of patient entitlements, and NHS decision makers may need to balance the need to comply with performance metrics against the budgetary limits that have been set.

These variations in explicitness of the HBP may, to a large extent, reflect limitations in the evidence on the effectiveness and appropriateness of certain treatments. Such limitations suggest that there may be a need to adopt a nuanced approach to setting an explicit HBP. At one extreme, when the quality of information about the costs and benefits of a treatment is good, and there is little heterogeneity in patient needs and preferences, it may be possible to make very clear statements about entitlements. For many treatments, where there is less reliable information, it may be necessary to allow “exceptions” from usual practice, which require an explicit statement or request from the clinician. Furthermore, theory suggests that treatments for which evidence is weak should be subject to a more demanding cost-effectiveness threshold [39]. Of course, whatever degree of hardness is adopted in an HBP specification, it will always be important to have an accurate assessment of its likely budgetary impact.

It is noteworthy that many recent initiatives in England are seeking to impose greater uniformity through guidelines, performance measures, and payment mechanisms. At the same time, there have been some moves toward increased local autonomy, for example, in the devolution of responsibilities and funding to certain cities such as Manchester. These apparently conflicting developments reflect the continuing tension between uniformity and local flexibility found in most health systems. Yet it is important to note that there are efforts to define health packages, often in the form of “essential” levels of services, even in decentralized health systems such as those found in Italy, Finland, and Sweden. There is a need for a clearly specified national HBP so that local decision makers can be held properly to account for their choices.

Thus, the fact that there is no formal HBP in England should not be interpreted as a suggestion that seeking to create such a package is infeasible or undesirable. Many of the policy problems confronted by NHS policymakers would to some extent be eased by the creation of a more explicit HBP. There are, however, many practical and political constraints to pursuing greater clarity, and there will always be a need to retain some flexibility in the services made available. Perhaps the biggest challenge to creating an explicit HBP is the concern that it would create unaffordable entitlements to care, and conflict with the local flexibility needed to adhere to budgets. This concern, however, may arise more from the unaffordable contents of the package rather than from the principle of setting out entitlements clearly.

As health systems seek to make a transition toward UHC, they must confront the issue of whether and how to establish an explicit benefits package, which sets out the treatments and services to which beneficiaries can secure access. Arrangements must also be put in place to ensure adequate quality of the services contained in the package. English policymakers have traditionally shied away from explicit specification of an HBP for various reasons. There are, nevertheless, signs that they may be moving toward an “intelligent” HBP, specified through instruments such as the NICE essential medicines list, broader treatment guidelines, performance measures, and payment mechanisms. The key challenge is to maintain an appropriate balance between clarity (when the evidence warrants) and flexibility (when it does not). Although there are important reasons why setting out an explicit HBP may be technically, administratively, and politically difficult, we suggest that, on the basis of the English experience, the difficulties that arise from failing to set out the HBP far outweigh any advantages, and that health systems should consider moving toward an explicitly specified HBP as an essential element of their UHC plans.

## Figures and Tables

**Table 1:** Types of NICE guidance [40]

NICE guidance takes several forms:
*NICE guidelines* make evidence-based recommendations on a wide range of topics, from preventing and managing specific conditions, improving health and managing medicines in different settings, to providing social care to adults and children, and planning broader services and interventions to improve the health of communities. These aim to promote integrated care where appropriate, for example, by covering transitions between children’s and adult services and between health and social care. *Technology appraisals* assess the clinical and cost-effectiveness of health technologies, such as new pharmaceutical and biopharmaceutical products, but also include procedures, devices, and diagnostic agents. This is to ensure that all NHS patients have equitable access to the most clinically and cost-effective treatments that are viable. *Medical technologies and diagnostics guidance* help to ensure that the NHS is able to adopt clinically and cost-effective technologies rapidly and consistently. *Interventional procedures guidance* recommends whether interventional procedures, such as laser treatments for eye problems or deep brain stimulation for chronic pain, are effective and safe enough for use in the NHS.

NHS, National Health Service; NICE, National Institute for Health and Care Excellence.
